# Age at First Full-term Pregnancy and Other Reproductive Factors Are Associated with Mammographic Breast Density in Postmenopausal Women: A Study in Flanders, Belgium

**DOI:** 10.1158/2767-9764.CRC-24-0561

**Published:** 2025-02-07

**Authors:** Magda J. Vandeloo, Eliane Kellen, Carolyn Y. Fang, Eric A. Ross, Liesbeth Vancoillie, Liesbeth M. Bruckers, Kristof Y. Neven, Esmée M. Bijnens, Tim S. Nawrot, Chantal Van Ongeval

**Affiliations:** 1 Department of Imaging & Pathology, University of Leuven, Leuven, Belgium.; 2 Centre for Environmental Sciences, Hasselt University, Hasselt, Belgium.; 3 Cancer Prevention and Control, Fox Chase Cancer Center-Temple Health, Philadelphia, Pennsylvania.; 4 LUCK, Campus St Rafael, University Hospital Leuven, Leuven, Belgium.; 5 Centre of Cancer Detection, Bruges, Belgium.; 6 Biostatistics and Bioinformatics Facility, Fox Chase Cancer Center-Temple Health, Philadelphia, Pennsylvania.; 7 Department of Radiology, University Hospital Leuven, Leuven, Belgium.; 8 I-BioStat, Data Science Institute, Hasselt University, Hasselt, Belgium.; 9 Faculty of Science/Department of Environmental Sciences, Open University Heerlen, Heerlen, the Netherlands.; 10 Department of Public Health and Primary Care, University of Leuven, Leuven, Belgium.

## Abstract

This study evaluated the association between age at first full-term pregnancy (FFTP) and mammographic breast density (MBD) in postmenopausal women. A total of 1,034 women, ages 50 to 69 years, were recruited from the Flemish (Belgium) population–based breast cancer screening program. Participants completed a questionnaire on lifestyle and reproductive factors. From mammography, we assessed the percentage glandular tissue of the total breast volume (GLAND), the volumetric breast density (VBD), and the Breast Imaging Reporting and Data System (BI-RADS) density classification. For statistical analysis, we used the piecewise linear regression model. The average age at FFTP was 26.1 years. Among women with an FFTP > **25.7** years, an each year increase in FFTP age was associated with a **1.17%** increase in GLAND [95% confidence interval (CI), 0.20%–2.46%; *P* = 0.041] and a **1.45%** increase in VBD (95% CI, 0.18%–2.75%; *P* = 0.026). Similarly, the odds of a higher BI-RADS classification increased by **5.0%** (95% CI, 0.0%–11.0%; *P* = 0.059) for each year increase in FFTP age after 25.7 years. For every year delay in age at menarche, a 2.48% higher GLAND (95% CI, 0.43%–4.57%; *P* = 0.017) and a 2.45% higher VBD (95% CI, 0.38%–4.56%; *P* ≤ 0.020) were observed. Ever use of oral contraceptive resulted in a 12.24% decrease in GLAND (95% CI, −20.90% to −2.63%; *P* = 0.014) and a 13.48% decrease in VBD (95% CI, −22.1% to −3.91%; *P* = 0.007). MBD is significantly higher when FFTP takes place after 25.7 years. Later age at menarche is associated with higher MBD, whereas ever use of oral contraceptive is associated with lower MBD at postmenopause.

**Significance:**

We consider our findings to be highly novel, and to the best of our knowledge, our study is one of the first to investigate associations between age at FFTP and three markers of MBD (GLAND, VBD, and BI-RADS), uncovering that MBD is significantly higher when FFTP occurs after 25.7 years.

## Introduction

Breast cancer is the most commonly diagnosed cancer among women worldwide, with an estimated 2.3 million new cases in 2020 and an increasing incidence (1). Breast cancer is the second leading cause of cancer-related mortality (2). Belgium has the world’s highest incidence of breast cancer (1), with an age-standardized rate of 107.0 per 100,000 person-years. Within Belgium, the northern region Flanders in particular has the highest incidence, with an age-standardized rate of 110.5 per 100,000 person-years (3).

Reproductive factors have been shown to be associated with breast cancer. Among them are age at menarche, age at first full-term pregnancy (FFTP), number of pregnancies, breastfeeding, use of oral contraceptives (OC), age at menopause, and use of hormones during menopause (4–6). Importantly, an FFTP that occurs at or before the age of 25 years has been shown to reduce a woman’s lifetime breast cancer risk by as much as 38% to 50%, and the risk is further reduced by another 7% for each subsequent pregnancy. In contrast, women who had their FFTP after the age of 30 have an increased breast cancer risk (2, 7). The biological and molecular mechanisms by which FFTP produces protection against breast cancer are due to the activation of genes controlling the process of chromatin remodeling (8).

Mammographic breast density (MBD) had been identified as a strong risk factor of breast cancer for the first time over 35 years ago by Wolfe (9) and was confirmed by subsequent studies (10–13). In a literature review by Houssami and Kerlikowski (14), the link between mammographic percentage density and dense area (DA) and breast cancer risk has been highlighted. Overall, MBD seems to represent a general marker of breast cancer risk that is not specifically in the breast with the cancer or at the quadrant location of the greatest density (15). Other studies reported that an association between MBD and risk may be the result of not only reproductive factors but also environmental factors and exposure to the local environment such as air purity, particulate materials, and other pollutants (16, 17).

MBD is based on the percentage of dense tissue, specifically stromal, epithelial, and adipose tissue, observed on a mammogram of the entire breast. Commonly, MBD is subjectively measured by a visual assessment of a mammogram. The most commonly used visual-based density evaluation tool is the Breast Imaging Reporting and Data System (BI-RADS; ref. 18), which is routinely reported for a large proportion of mammograms in the United States and Europe. This BI-RADS classification changed from a visual qualitative assessment in the fourth edition to a more sensitive evaluation of the mammogram in the fifth edition (18, 19).

However, due to the subjective nature of these classifications, considerable inter- and intrarater variability exist. As digitizing film-screen mammography has become possible, followed by full-field digital mammography, semiautomated and automated methods of objectively assessing density have been increasingly utilized, including the semiautomated area-based Cumulus methods and volumetric methods, such as Volpara, Quantra, and the single-energy X-ray absorptiometry method (19).

To date, many of the studies investigating the associations among reproductive factors, MBD, and breast cancer risk have used radiologists’ BI-RADS assessments of MBD (20–23). Studies utilizing BI-RADS assessments are more subjective and less precise than those utilizing objective measurements of MBD, such as Volpara, but they have the advantage of larger and more diverse patient populations as well as follow-up information on the subsequent development of breast cancer. However, to date, very few studies have examined whether early FFTP might be associated with lower MBD, objectively measured in postmenopausal women (24). Thus, the aim of this study is to determine the role of age at FFTP, along with other reproductive factors, and MBD, objectively measured in postmenopausal women participating in a regional screening program.

## Materials and Methods

### Study population and data collection

**Figure 1:**
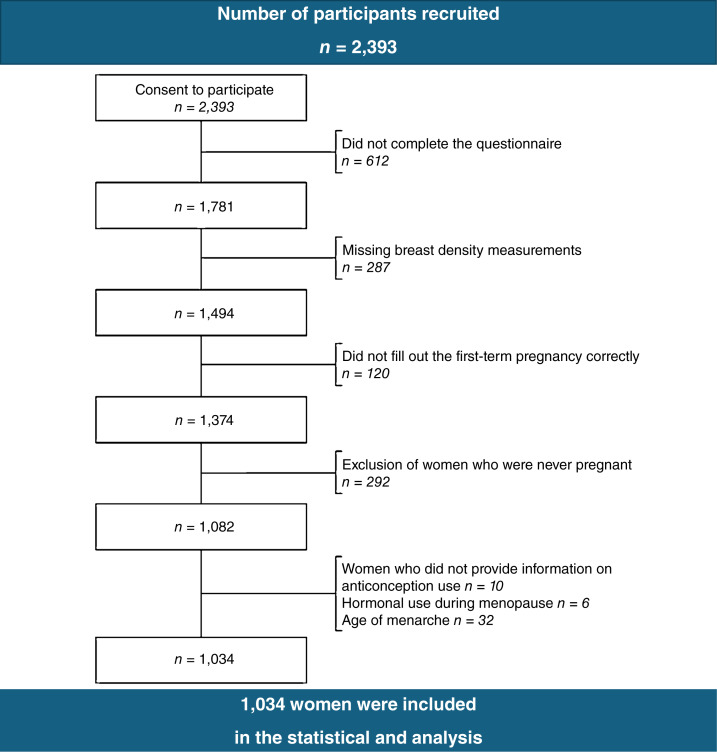
Selection process indicating the number of women recruited and excluded during the process of collection and data analysis.

In this cross-sectional study, women, ages 50 to 69 years, who were participating in the Flemish (Belgium) population–based breast cancer screening program were requested to collaborate during their visit at the mammography department at the University Hospital Leuven (UZ Leuven) or at its mobile unit. From all the participants, we obtained written informed consent, and our study has been conducted according to the ethical guidelines expressed in the “Declaration of Helsinki” (25). A self-administered questionnaire (SAQ) containing 218 questions capturing detailed information on lifestyle, reproductive factors, and environmental exposure was requested to be completed. The SAQ of our study was divided into four chapters related to the major periods in the life of women: (i) the childhood period (around the age of 6 years); (ii) the period of adulthood (around the age of 20 years); (iii) the period of perimenopause (around the age of 35 years); and (iv) the period of menopause (around the age of 50 years). Women were asked to report information on, e.g., year of birth, age at menarche, parity, age at FFTP, breastfeeding, number of pregnancies, family history of breast cancer, personal history of benign breast disease, hormonal contraceptive use [oral contraceptive (OC)], nutritional factors, alcohol consumption, body mass index (BMI), smoking habits, and menopausal hormone therapy use [hormone replacement therapy (HRT)]. A reminder to complete the SAQ was sent out weekly to the nonrespondents in order to promote participation, starting from the first week until 1 month after obtaining informed consent. The data were registered in a central database using “Qualtrics” software. From the 2,393 participants that were enrolled in the study and provided informed consent, 1,781 women (74.4%) completed the SAQ. After exclusion of participants with missing data, 1,034 respondents (43.2%) remained for the statistical analysis (Fig. 1). The mean age of the participants at MBD measurements was 58.6 years (SD = 5.6; Table 1).

**Table 1:** Sociodemographic characteristics. Stratification on age at FFTP was made based on the results of the NLIN procedure in SAS.

Characteristic	FFTP ≤ 25.7 (*N* = 504)	FFTP > 25.7 (*N* = 530)	Overall (*N* = 1,034)
Age at MBD measurement, (years)	60.1 (5.3)	57.1 (5.4)	58.6 (5.6)
Use of hormones during menopause			
No	390 (77.4%)	442 (83.4%)	832 (80.5%)
Yes	114 (22.6%)	88 (16.6%)	202 (19.5%)
Number of live-born children			
1 child	105 (20.8%)	106 (20.0%)	211 (20.4%)
2 children	209 (41.5%)	209 (39.4%)	418 (40.4%)
3 or more children	190 (37.7%)	215 (40.6%)	405 (39.2%)
Regular exercise at 50 years of age			
No	182 (36.1%)	175 (33.0%)	357 (34.5%)
Yes	322 (63.9%)	355 (67.0%)	677 (65.5%)
Education level^a^			
High	174 (34.5%)	364 (68.7%)	538 (52.0%)
Middle	237 (47.0%)	136 (25.7%)	373 (36.1%)
Low	76 (15.1%)	16 (3.0%)	92 (8.9%)
Self-reported smoking habit			
Never smoker	272 (54.0%)	300 (56.6%)	572 (55.3%)
Current or past smoker	232 (46.0%)	230 (43.4%)	462 (44.7%)
Current BMI (kg/m^2^)^b^			
Underweight and normal	210 (41.7%)	270 (50.9%)	480 (46.4%)
Overweight	189 (37.5%)	176 (33.2%)	365 (35.3%)
Obese	105 (20.8%)	84 (15.8%)	189 (18.3%)
Use of a contraceptive pill			
No	55 (10.9%)	32 (6.0%)	87 (8.4%)
Yes	449 (89.1%)	498 (94.0%)	947 (91.6%)
Ever breastfed			
No	259 (51.4%)	183 (34.5%)	442 (42.7%)
Yes	245 (48.6%)	347 (65.5%)	592 (57.3%)
Age at the menarche (years)	13.0 (1.5)	13.1 (1.4)	13.1 (1.4)
Age at the FFTP (years)	22.7 (2.1)	29.3 (3.2)	26.1 (4.3)

Data are presented as mean (SD) for numerical variables and as *n* (%) for categorical variables.

a “Education level” was coded as low (no diploma or primary school), middle (high school), and high (college or university degree). Data are available for 487, 516 and 1,003 participants, respectively.

b “Current BMI (kg/m^2^)” was divided into three categories according to the World Health Organization: (i) underweight and normal (BMI = below 18.5–24.9), which is the referent group; (ii) overweight (BMI = 25–29.9); and (iii) obese (BMI = 30 or more). Data are available for 504, 530, and 1,034 participants, respectively.

### Demographic and reproductive variables: Definition and categories

To analyze the association between “education” and MBD, we categorized this variable into three different groups: (i) low = no diploma or primary school; (ii) middle = high school; and (iii) high = college or university degree. To examine the relationship between the reproductive factors “use of a contraceptive pill” and “use of hormones during menopause” and MBD, we used the parameter (overall answer of the women) ever used “yes” compared with “no.” The variable “number of live-born children” was divided into three categories: (i) one child (referent group), (ii) two children, and (iii) three or more children. To investigate whether there was an association between breastfeeding and MBD, we used the parameter (overall answer of the women) ever breastfed “yes” compared with “no.” The variable “current BMI (kg/m^2^)” was divided into three categories, according to the World Health Organization (https://www.who.bmi int/data): (i) underweight and normal (BMI = below 18.5–24.9; referent group); (ii) overweight (BMI = 25–29.9); and (iii) obese (BMI = 30 or more). To define the variable “regular exercise at the age of 50,” women were asked whether they perform exercise on a regular basis for at least 30 minutes/week, i.e., walking, bicycling, or even working in the garden. We classified the variable “self-reported smoking habit” into two groups: (i) never-smoker and (ii) current or past smoker.

### Ethical aspects

All women who were willing to participate were required to provide written informed consent. All participants were able to understand the Dutch language. Any personal information was encrypted. The study protocol was approved by the Ethical Committee of the Leuven University (Belgium) and the UZ Leuven (Belgium) and was carried out in accordance with the “Declaration of Helsinki” (25).

### Objective assessment of MBD

We used the mammography raw data retrieved from the hospital mammography system servers as an input to the software program Volpara (VolparaDataManager). Volpara uses a combination of X-ray physics and machine learning to generate an accurate and objective volumetric measure of breast composition. Volpara’s TruDensity algorithm automatically assesses the volumetric breast density (VBD) percentage of each mammogram on a continuous scale; a typical VBD range is 2% to 35%. This differentiates each woman on a continuum of density. These scores correlate with the BI-RADS fifth edition density categories (18, 26). This is the most commonly used categorization system for assessing MBD. BI-RADS classifies MBD into four categories: entirely fatty (<25% of dense tissue; Table 2, class 1); scattered fibroglandular densities within the breast (25%–50% dense tissue; Table 2, class 2); heterogeneously dense (50%–75% dense tissue; Table 2, class 3); and extremely dense (>75% of dense tissue; Table 2, class 4; ref. 18). Additionally, the percentage glandular tissue of the total breast volume (GLAND) and the BI-RADS density classification were also extracted from Volpara software. These three parameters were chosen as the clinical translation of MBD.

**Table 2:** MBD characteristics. Stratification on age at FFTP was made based on the results of the NLIN procedure in SAS.

Characteristic	FFTP ≤ 25.7 (*N* = 504)	FFTP > 25.7 (*N* = 530)	Overall (*N* = 1,034)
GLAND (cm³)	15.1 (9.76)	16.9 (10.6)	16.0 (10.2)
VBD (%)	7.35 (4.7)	8.24 (5.1)	7.80 (5.0)
BI-RADS			
Class 1	66 (13.1%)	60 (11.3%)	126 (12.2%)
Class 2	231 (45.8%)	223 (42.1%)	454 (43.9%)
Class 3	163 (32.3%)	175 (33.0%)	338 (32.7%)
Class 4	44 (8.7%)	72 (13.6%)	116 (11.2%)

Data are presented as mean (SD) for numerical variables and as *n* (%) for categorical variables.

### Statistical analysis

Database management and statistical analysis were performed with the SAS software version 9.4 (SAS Institute). Mean ± SD is given for the continuous variables GLAND and VBD and the proportion for the categorical variable BI-RADS. The parameters GLAND and VBD were transformed according to the natural logarithm to improve normality. Based on the visual inspection of the association between the log (GLAND) and log (VBD) and the FFTP, we opted for a piecewise linear regression (PLR) model using the nonlinear regression (NLIN) procedure in SAS (version 9.4, SAS Institute; RRID: SCR_008567). The PLR model produces multiple linear segments and joined at a breakpoint. The model was adjusted for *a priori* chosen variables: age at menarche, ever use of OC, ever use of HRT, age at screening, number of live-born children, current BMI, ever breastfed, regular exercise at the age of 50, educational level, and self-reported smoking habit. In a secondary analysis, the model was simplified via the backward selection procedure by excluding variables with *P* value > 0.10 in the full model. For the parameters GLAND and VBD, results are presented as % change [with 95% confidence intervals (CI)] by transforming the β from the model with the Euler number (e^β^). For the BI-RADS, we used ordinal logistic regression analysis. All hypothesis tests were two-sided with a 5% type I error (Supplementary Tables S1–S3).

### Data availability

Raw data for this study were generated at the UZ Leuven. Derived data supporting the findings of this study are available from the corresponding author upon request.

## Results

During the period from January 8, 2018, through January 28, 2020, 2,393 participants were enrolled in the study. Of the 2,393 participants who provided informed consent, 1,781 (74.4%) completed the SAQ. After exclusion of participants with missing data, 1,034 (43.21%) respondents remained for the statistical analysis (Fig. 1).

### Population characteristics

Sociodemographic characteristics of the 1,034 participating women are presented in Table 1. The mean age at MBD measurements was 58.6 years (SD = 5.6). Most women had at least two live-born children (79.6%) and obtained at least a college or university degree (52.0%). Only 87 (8.4%) of the participants indicated to have never used an OC. A majority had ever breastfed at least one of their children (57.3%). The menarche was reached at the mean age of 13.1 years (SD = 13.1). The women had their FFTP at 26.1 years of age (SD = 4.3).

**Figure 2:**
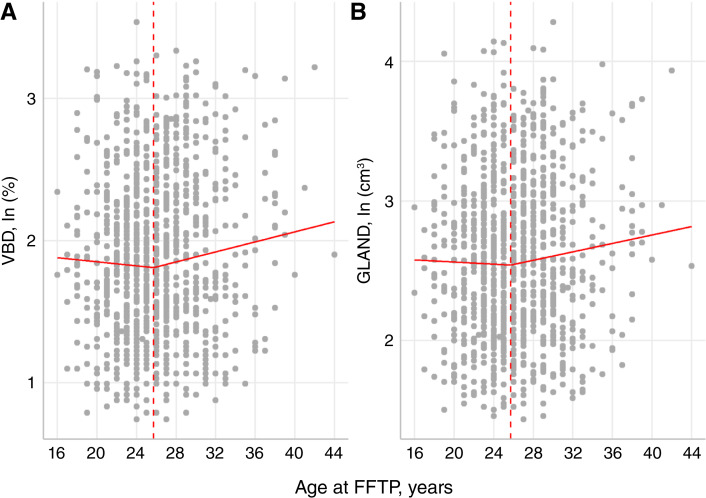
Correlation between MBD and age at FFTP. Plots (**A** and **B**) show the association of the VBD [in ln (%)] and GLAND [in ln (cm³)] with age at FFTP (in years). Breakpoint is indicated as a red dotted line at 25.7 years.

Based on the optimal breakpoint of the NLIN model at the FFTP age of 25.7 years (95% CI, 22.3–29.1) illustrated in Fig. 2, we stratified the following tables (Table 1 to and including Table 5) into “FFTP ≤25.7 years” and “FFTP > 25.7 years”.

Table 2 shows the MBD characteristics of the study population. The average value for GLAND was 16.0 cm³ (SD = 10.2 cm³) and for VBD was 7.8% (SD = 5.0%). Most women had the second lowest BI-RADS classification (class 2, 43.9%). A total of 126 (12.2%) women had a class 1 BI-RADS classification, whereas 116 (11.2%) women received a class 4 result.

### Association between the age at FFTP and MBD characteristics after menopause

The NLIN model showed an optimal breakpoint at the FFTP age of **25.7** years (95% CI, 22.3–29.1; Fig. 2). This means that there is a significant difference in the association between MBD and age at FFTP above the breakpoint. Both Fig. 2A and B shows that women with an FFTP before the age of 25.7 years have no statistically significant association (β = −1.3%; 95% CI, −3.3%–0.7%; benefit) of being pregnant earlier for their GLAND or VBD. However, after the age of 25.7, there was a significant increase in these densities with increased age at FFTP. After adjustment for *a priori* chosen variables, both GLAND and VBD were positively associated with the age at FFTP after 25.7 years (Tables 3 and 4, respectively). An each year increase in age at FFTP was associated with an increase of **1.17%** (95% CI, 0.20–2.46; *P* = 0.041) in GLAND and **1.45%** (95% CI, 0.18–2.75; *P* = 0.026) in VBD. No significant effects were observed between either MBD parameter and age at FFTP below 25.7 years. In the multivariate NLIN models, age at menarche and hormonal use during menopause were associated with a higher VBD and GLAND, whereas current BMI of overweight and obese women, age at measurement, use of a contraceptive pill, and parity were linked with a lower VBD and GLAND.

**Table 3:** Results from the PROC NLIN model for the **GLAND**.

Variable	Estimate	95% CI LL	95% CI UL	*P* value
FFTP, *+1 year*				
*≤25.7*	−0.99%	−2.96%	1.01%	0.33
*>25.7*	**1.17%**	0.20%	2.46%	0.041
Age at MBD measurement, *+1 year*	−1.15%	−1.69%	−0.61%	<0.0001
Age at menarche, *+1 year*	2.48%	0.43%	4.57%	0.017
Use of a contraceptive pill, *yes compared with no*	−12.24%	−20.90%	−2.63%	0.014
Use of hormones during menopause, *yes compared with no*	8.13%	0.54%	16.30%	0.035
Current BMI (kg/m^2^)				
Underweight and normal	Referent			
Overweight	−38.49%	−42.28%	−34.43%	<0.0001
Obese	−58.48%	−61.64%	−55.04%	<0.0001
Number of live-born children				
1 child	Referent			
2 children	−3.55%	−10.68%	4.15%	0.363
3 or more children	−9.01%	−15.80%	−1.68%	0.017

Multiplicative estimates provided are calculated according to e^β^ for the given unit change.

The multivariate PLR model was adjusted for *a priori* chosen variables: age at menarche, ever use of OC, ever use of HRT, age at screening, number of live-born children, and current BMI.

Abbreviations: LL, lower limit; UL, upper limit.

**Table 4:** Results from the PROC NLIN model for the VBD.

Variable	Estimate	95% CI LL	95% CI UL	*P* value
FFTP, *+1 year*				
*≤25.7*	−1.20%	−3.19%	0.81%	0.24
*>25.7*	**1.45%**	0.18%	2.75%	0.026
Age at MBD measurement, *+1 year*	−1.17%	−1.72%	−0.62%	<0.0001
Age at menarche, *+1 year*	2.45%	0.38%	4.56%	0.020
Use of a contraceptive pill, *yes compared with no*	−13.48%	−22.10%	−3.91%	0.007
Use of hormones during menopause, *yes compared with no*	6.89%	−0.70%	15.05%	0.076
Current BMI (kg/m^2^)				
Underweight and normal	Referent			
Overweight	−37.12%	−41.05%	−32.94%	<0.0001
Obese	−57.80%	−61.05%	−54.27%	<0.0001
Number of live-born children				
1 child	Referent			
2 children	−3.78%	10.96%	3.99%	0.336
3 or more children	−10.17%	−16.94%	−2.84%	0.007

Multiplicative estimates provided are calculated according to e^β^ for the given unit change.

The multivariate PLR model was adjusted for *a priori* chosen variables: age at menarche, ever use of OC, ever use of HRT, age at screening, number of live-born children, and current BMI.

Abbreviations: LL, lower limit; UL, upper limit.

In the following tables “Tables 3–5” and the “Supplementary Tables S1–S3,” only the variables (listed in Table 1) with a significant association with regard to the three parameters of MBD are described. We found no significant relationship between the MBD markers and regular exercise at the age of 50, education level, smoking, and breastfeeding after correcting for age at MBD measurement and age at FFTP.

To ensure comparability between the MBD parameters, we stratified the age based on the optimal breakpoint of the NLIN model into “≤25.7 years” and “>25.7 years” in the ordinal logistic regression model of BI-RADS. After adjusting for covariates, an increase of 1 year in FFTP above the age of 25.7 years was associated with an OR of **1.05** (95% CI, 1.00–1.11; *P* = 0.059). As with the previous MBD parameters, no significant association was found at an FFTP age below 25.7. For the covariates, only the current BMI of obese women was significantly associated with a lower BI-RADS score for participants in either FFTP group. For participants with an FFTP ≤ 25.7 years, only age at menarche showed a higher odds at a higher BI-RADS classification. Participants with an FFTP > 25.7 years had lower odds for a higher BI-RADS classification for the age at MBD, hormonal use during menopause, and parity, whereas the use of a contraceptive pill showed higher odds for a higher BI-RADS class.

### Conclusion of results

In this sample, the average age at FFTP was 26.1 years (5th–95th percentile: 20–34). The PLR model estimated the breakpoint in our analysis at an FFTP age of **25.7 years** (95% CI, 22.3–29.1; Fig. 2). For women with an FFTP age younger than 25.7 years, the association between GLAND or VBD and FFTP was not statistically significant. For woman with an FFTP age above 25.7 years, an each year increase in FFTP age was associated with a **1.17%** increase in GLAND (95% CI, 0.20%–2.46%; *P* = 0.041) and a **1.45%** increase in VBD (95% CI, 0.18%–2.75%; *P* = 0,026; Tables 3 and 4).

The analysis of the BI-RADS showed similar results, with the odds of belonging to a higher BI-RADS classification increased by **5.00%** (95% CI, 0.0%–11.0%; *P* = 0.059) for an each year increase in FFTP age after the age of 25.7 years (Table 5). Among other reproductive factors such as age at menarche, for every year delay, there was a 2.48% higher GLAND (95% CI, 0.43%–4.57%; *P* = 0.017) and a 2.45% higher VBD (95% CI, 0.38%–4.56%; *P* ≤ 0.020), whereas the ever use of OC resulted in a 12.24% decrease in GLAND (95% CI, −20.90% to −2.63%; *P* = 0.014) and a 13.48% decrease in VBD (95% CI, −22.10% to −3.91%; *P* = 0.007; Tables 3 and 4).

**Table 5:** Results from the ordinal logistic regression model for BI-RADS. The model was stratified based on the FFTP results from the GLAND and VBD models: “≤25.7 years” and “>25.7 years.”

	For an FFTP ≤ 25.7 years (*n* = 504)				For an FFTP > 25.7 years (*n* = 530)			
Variable	OR	95% CI LL	95% CI UL	*P* value	OR	95% CI LL	95% CI UL	*P* value
FFTP, *+1 year*	0.97	0.89	1.06	0.53	**1.05**	1.00	1.11	0.059
Age at MBD measurement, *+1 year*	0.97	0.94	1.00	0.97	0.96	0.93	0.99	0.004
Age at menarche, *+1 year*	1.19	1.05	1.34	0.0048	1.08	0.96	1.22	0.19
Use of a contraceptive pill*, yes compared with no*	1.20	0.69	2.09	0.52	1.93	0.96	3.87	0.066
Use of hormones during menopause*, yes compared with no*	0.99	0.66	1.51	0.98	0.63	0.40	0.98	0.0415
Current BMI (kg/m^2^)								
Underweight and normal	Referent							
Overweight	0.17	0.11	0.25	0.26	0.20	0.14	0.30	0.87
Obese	0.04	0.02	0.07	<0.0001	0.04	0.02	0.07	<0.0001
Number of live-born children								
1 child	Referent							
2 children	0.93	0.59	1.47	0.45	0.84	0.54	1.32	0.66
3 or more children	0.66	0.41	1.05	0.11	0.61	0.39	0.95	0.0192

Data are presented as OR estimates and 95% Wald CIs.

The multivariate PLR model was adjusted for *a priori* chosen variables: age at menarche, ever use of OC, ever use of HRT, age at screening, number of live-born children, and current BMI.

Abbreviations: LL, lower limit; UL, upper limit.

## Discussion

The link between MBD and breast cancer risk is well documented (9, 10, 12, 14). However, the extent to which reproductive factors influence breast cancer risk through their effects on MBD and the degree to which they influence breast cancer risk through other pathways are unknown (27).

In the present cross-sectional study, we examined the association of age at FFTP and other reproductive factors with objectively measured MBD in women, ages 50 to 69 years, who were participating in the Flemish (Belgium) population–based breast cancer screening program. We showed that MBD significantly increased when FFTP takes place after 25.7 years. Furthermore, we found that later age at menarche is associated with higher MBD, whereas ever use of an OC is associated with lower MBD at postmenopause. We found no significant relationship between the MBD markers and regular exercise at the age of 50, education level, smoking, and breastfeeding after correcting for age at MBD measurement and age at FFTP.

### Comparison with previous studies

Although published literature provides us with insights into the biological and molecular bases of FFTP and the mechanisms underlying protection against breast cancer (5, 8, 28–30), very few studies have investigated whether early FFTP might be associated with lower MBD in postmenopausal women. According to the work by Russo (31, 32), the pattern of differentiation and involution of the breast that determine MBD and therefore an increased risk of developing breast cancer at menopause are closely related to one another. Reproductive history is consistently and reliably associated with the risk of developing breast cancer (33). A recent systematic review (24) examining the association between age at FFTP and objectively measured MBD in postmenopausal women found that 6 of 12 included studies reported an association between older age at FFTP and higher MBD (34–39).

In the study of Yaghjyan and colleagues (35), 4,110 cancer-free women within the Nurses’ Health Study and Nurses’ Health Study II cohorts were included. Percent breast density, absolute dense, and non-DAs were measured from digitized mammography film images with computerized techniques. Among parous postmenopausal women, older age at FFTP was positively associated with percent density (PD; β = 0.03; 95% CI, 0.01–0.05) and inversely associated with non-DA (β = −0.10; 95% CI, −0.13 to −0.06; ref. 35).

However, only the study of Vandeloo and colleagues (39) investigated associations between age at FFTP and the three markers of MBD (GLAND, VBD, and BI-RADS). The remaining six studies in this review either reported no relationship or revealed an inverse association between MBD and older age at FFTP. The present study is consistent with six previously published studies that reported an association between older age at FFTP and higher MBD. Although some previous studies found that MBD increases with age at FFTP, they have not found that this increase is restricted to FFTP after 25.7 years of age (24, 36). However, due to differences across studies, no conclusions could be drawn with regard to the links among age at FFTP, MBD, and breast cancer risk.

When comparing the statistically significant association between later age at menarche and higher MBD found in our study with findings from prior studies, we can conclude that the observed direction of the association with MBD is mostly similar (36, 38, 40, 41). Alexeeff and colleagues showed that older age at menarche was associated with higher PD, such that women whose age at menarche was ≥16 years were estimated to have a 2.9% higher PD and 2.4 cm^2^ higher DA than women whose age at menarche was <10 years (36). Furthermore, within one of the largest international studies to date, consisting of populations with different ethnic backgrounds, Ward and colleagues found that later age at menarche was positively associated with both percent and absolute DAs. As increased MBD is associated with increased breast cancer risk, the paradoxical positive association between later menarche and MBD is not well understood (41). Ghadge and colleagues summarized studies reporting associations between timing of puberty and MBD and found that most studies reported an association between later age at menarche and MBD but not all studies. Thus, the protective effect of later age at menarche on breast cancer risk is not likely mediated through MBD (42). However, in a large cross-sectional study of 8,460,928 women, ages >40 years, results showed that earlier age at menarche (<15 vs. ≥15; adjusted OR, 1.18; 95% CI, 1.17–1.18) was associated with dense breasts (43). In contrast, other studies did not show an association between age at menarche and MBD (44, 45).

In our study, we found that ever use of an OC was associated with lower MBD at postmenopause. When comparing with previous studies among postmenopausal women, findings of one recent large cross-sectional study were consistent with our study. Kim and colleagues (43) suggested that women with higher BMI and the use of OCs were more likely to have nondense breasts. In contrast, Hunt and colleagues (46) concluded that the initiation of a combined OC was associated with an increase in BI-RADS category, although this might be a transient effect, whereas other studies found no association between ever use of OCs and MBD (44, 45). These conflicting findings about the association between the use of OCs and MBD might be explained by heterogeneity in sample sizes, ethnicity, and methodology utilized.

Our results on the correlation of the number of live-born children and the use of hormones during menopause with MBD in postmenopausal women are consistent with previous reports (33–35, 40, 43, 44). The findings of Yaghjyan and colleagues suggest that among postmenopausal parous women, a greater number of live-born children was inversely associated with both PD (β = −0.07; 95% CI, −0.12 to −0.02) and absolute DA (β = −0.14; 95% CI, −0.21 to −0.06; ref. 35). Furthermore, the results of a recent systematic review of Azam and colleagues (47) showed that MBD was significantly increased in ever HRT users compared with never-users, with the highest increase in MBD observed among current HRT users. These results were also consistent with findings of Rice and colleagues (40) showing that the current use of postmenopausal HRT was positively associated with MBD.

### Strengths and limitations

This study has several strengths and limitations. A key strength of the present study is that to the best of our knowledge, we have established for the first time the numerical factors for the importance of the age at FFTP by indicating how the three parameters of MBD (GLAND, VBD, and BI-RADS), measured with the latest quantitative and objective method, change with age at FFTP. Importantly, these numerical factors increase for every year that FFTP is delayed after 25.7 years of age. Although age at FFTP explains only a small amount of variability in MBD, these quantitative assessments provide valuable insights. When considered alongside other established risk factors, such as MBD itself, this information may contribute to refining models that identify women at increased risk of breast cancer, which could have implications for screening and prevention recommendations. Another strength of this study is that all MBD measurements were conducted objectively using Volpara software, a sophisticated and widely accepted objective method. Among the various available software applications, Volpara is highly accepted in many radiology centers because of its strong agreement with the visual BI-RADS assessment by radiologists (48). Discrepancies between assessment by radiologists and automated software arise most frequently at the transition of BI-RADS 2 to 3 and 3 to 4; in these situations, an objective measurement is most helpful and much more reliable. In case of difference between left and right breasts, Volpara can help resolve these discrepancies.

However, we also acknowledge that some limitations to the present study should be considered. A limitation of the current study is its relatively modest sample size, although our sample is comparable with those of previous studies (34, 35). Furthermore, we examined a homogeneous population of women from a specific area of Flanders. It would be interesting for future studies to expand our study population not only to other regions of Belgium that are more heterogeneous than our present population but also to other countries and racial groups. Another limitation of the current cross-sectional study is that recall bias for selected reproductive variables in postmenopausal women is possible. Furthermore, we recognize that women who are younger at the time of FFTP are likely to have more children and to breastfeed compared with women who were older at FFTP and have had only one or two children. BMI clearly increases with the number of pregnancies, and this factor is also known to be associated with lower MBD. Other important variables may affect the association between the age of FFTP and MBD. Further research will be needed to determine their overall impact.

In conclusion, we consider our findings to be highly novel, and to the best of our knowledge, our study is one of the first to investigate associations between the age at FFTP and three markers of MBD (GLAND, VBD, and BI-RADS), suggesting that MBD is significantly higher when FFTP occurs after 25.7 years of age. The present study provides valuable data to inform the design of future longitudinal studies to corroborate that the increased MBD observed with increasing age at FFTP is associated with breast cancer development. If confirmed, these findings may enable early identification of the subgroup of women at increased risk of developing breast cancer who would benefit from breast cancer prevention approaches.

## Supplementary Material

Table S1 Table S1: Results from the univariate analysis for the glandular tissue (GLAND). For FFTP, we used the proc NLIN procedure, for the others a GLM. Multiplicative estimates provided are calculated according to eβ for the given unit change

Table S2 Table S2: Results from the univariate analysis for the volumetric breast density (VBD). For FFTP, we used the proc NLIN procedure, for the others a GLM. Multiplicative estimates provided are calculated according to eβ for the given unit change

Table S3 Table S3: Results from the univariate ordinal logistic regression model for BI-RADS. The model for FFTP was stratified based on the results from the GLAND and VBD models: ‘≤ 25.7 years’ and ‘> 25.7 years’. Data is presented as Odds Ratio (OR) estimates and 95% Wald confidence intervals

## References

[1] Sung H , Ferlay J, Siegel RL, Laversanne M, Soerjomataram I, Jemal A, . Global cancer statistics 2020: GLOBOCAN estimates of incidence and mortality worldwide for 36 cancers in 185 countries. CA Cancer J Clin 2021;71:209–49. 33538338 10.3322/caac.21660

[2] Subramani R , Lakshmanaswamy R. Pregnancy and breast cancer. Prog Mol Biol Transl Sci 2017;151:81–111. 29096898 10.1016/bs.pmbts.2017.07.006

[3] Belgian Cancer Registry . Cancer fact sheet breast cancer ICD10 : C50. 2021.Available from: https://kankerregister.org/sites/default/files/2024/Cancer_Fact_Sheet_Female%20Breast%20Cancer_2022.pdf.

[4] Kelsey JL , Gammon MD, John EM. Reproductive factors and breast cancer. Epidemiol Rev 1993;15:36–47. 8405211 10.1093/oxfordjournals.epirev.a036115

[5] Russo J , Balogh GA, Russo IH. Full-term pregnancy induces a specific genomic signature in the human breast. Cancer Epidemiol Biomarkers Prev 2008;17:51–66. 18199711 10.1158/1055-9965.EPI-07-0678

[6] Azubuike SO , Hayes L, Sharp L, McNally R. Reproductive factors and the risk of breast cancer among Nigerian women by age and oestrogen receptor status. Cancer Causes Control 2022;33:1401–12. 36190666 10.1007/s10552-022-01629-z

[7] MacMahon B , Cole P, Lin TM, Lowe CR, Mirra AP, Ravnihar B, . Age at first birth and breast cancer risk. Bull World Health Organ 1970;43:209–21. 5312521 PMC2427645

[8] Russo J , Santucci-Pereira J, de Cicco RL, Sheriff F, Russo PA, Peri S, . Pregnancy-induced chromatin remodeling in the breast of postmenopausal women. Int J Cancer 2012;131:1059–70. 22025034 10.1002/ijc.27323 PMC3350833

[9] Wolfe JN . Breast patterns as an index of risk for developing breast cancer. AJR Am J Roentgenol 1976;126:1130–7. 179369 10.2214/ajr.126.6.1130

[10] McCormack VA , dos Santos Silva I. Breast density and parenchymal patterns as markers of breast cancer risk: a meta-analysis. Cancer Epidemiol Biomarkers Prev 2006;15:1159–69. 16775176 10.1158/1055-9965.EPI-06-0034

[11] Huo CW , Chew GL, Britt KL, Ingman WV, Henderson MA, Hopper JL, . Mammographic density-a review on the current understanding of its association with breast cancer. Breast Cancer Res Treat 2014;144:479–502. 24615497 10.1007/s10549-014-2901-2

[12] Pettersson A , Graff RE, Ursin G, Santos Silva ID, McCormack V, Baglietto L, . Mammographic density phenotypes and risk of breast cancer: a meta-analysis. J Natl Cancer Inst 2014;106:dju078. 24816206 10.1093/jnci/dju078 PMC4568991

[13] Boyd NF , Martin LJ, Yaffe MJ, Minkin S. Mammographic density and breast cancer risk: current understanding and future prospects. Breast Cancer Res 2011;13:223. 22114898 10.1186/bcr2942 PMC3326547

[14] Houssami N , Kerlikowske K. The impact of breast density on breast cancer risk and breast screening. Curr Breast Cancer Rep 2012;4:161–8.

[15] Vachon CM , Brandt KR, Ghosh K, Scott CG, Maloney SD, Carston MJ, . Mammographic breast density as a general marker of breast cancer risk. Cancer Epidemiol Biomarkers Prev 2007;16:43–9. 17220330 10.1158/1055-9965.EPI-06-0738

[16] Huynh S , von Euler-Chelpin M, Raaschou-Nielsen O, Hertel O, Tjønneland A, Lynge E, . Long-term exposure to air pollution and mammographic density in the Danish Diet, Cancer and Health cohort. Environ Health 2015;14:31. 25879829 10.1186/s12940-015-0017-8 PMC4392475

[17] Yaghjyan L , Arao R, Brokamp C, O’Meara ES, Sprague BL, Ghita G, . Association between air pollution and mammographic breast density in the Breast Cancer Surveilance Consortium. Breast Cancer Res 2017;19:36. 28381271 10.1186/s13058-017-0828-3 PMC5382391

[18] American College of Radiology . ACR BI-RADS Atlas. 5th ed. Breast Imaging Reporting and Data System; 2013. ACR.

[19] Destounis S , Arieno A, Morgan R, Roberts C, Chan A. Qualitative versus quantitative mammographic breast density assessment: applications for the US and abroad. Diagnostics (Basel) 2017;7:30. 28561776 10.3390/diagnostics7020030 PMC5489950

[20] Alomaim W , O’Leary D, Ryan J, Rainford L, Evanoff M, Foley S. Subjective versus quantitative methods of assessing breast density. Diagnostics (Basel) 2020;10:331. 32455552 10.3390/diagnostics10050331 PMC7277954

[21] Kerlikowske K , Ichikawa L, Miglioretti DL, Buist DSM, Vacek PM, Smith-Bindman R, . Longitudinal measurement of clinical mammographic breast density to improve estimation of breast cancer risk. J Natl Cancer Inst 2007;99:386–95. 17341730 10.1093/jnci/djk066

[22] Nishiyama K , Taira N, Mizoo T, Kochi M, Ikeda H, Iwamoto T, . Influence of breast density on breast cancer risk: a case control study in Japanese women. Breast Cancer 2020;27:277–83. 31650498 10.1007/s12282-019-01018-6

[23] Riza E , Remoundos D-D, Bakali E, Karadedou-Zafiriadou E, Linos D, Linos A. Anthropometric characteristics and mammographic parenchymal patterns in post-menopausal women: a population-based study in Northern Greece. Cancer Causes Control 2009;20:181–91. 18814046 10.1007/s10552-008-9232-8

[24] Vandeloo MJ , Kellen E, Fang CY, Neven KY, Nawrot MJ, Van Ongeval C. Age first full-term pregnancy mammographic breast density postmenopausal women: a systematic review. Syst Rev 2023;44:1–13.

[25] World Medical Association . World medical association declaration of Helsinki: ethical principles for medical research involving human subjects. JAMA 2013;310:2191–4. 24141714 10.1001/jama.2013.281053

[26] van der Waal D , den Heeten GJ, Pijnappel RM, Schuur KH, Timmers JMH, Verbeek ALM, . Comparing visually assessed BI-rads breast density and automated volumetric breast density software: a cross-sectional study in a breast cancer screening setting. PLoS One 2015;10:e0136667. 26335569 10.1371/journal.pone.0136667 PMC4559403

[27] Rice MS , Bertrand KA, VanderWeele TJ, Rosner BA, Liao X, Adami HO, . Mammographic density and breast cancer risk: a mediation analysis. Breast Cancer Res 2016;18:94. 27654859 10.1186/s13058-016-0750-0 PMC5031307

[28] Russo J , Santucci-Pereira J, Russo IH. The genomic signature of breast cancer prevention. Genes (Basel) 2014;5:65–83. 24705287 10.3390/genes5010065 PMC3978512

[29] Gutiérrez-Díez PJ , Gomez-Pilar J, Hornero R, Martínez-Rodríguez J, López-Marcos MA, Russo J. The role of gene to gene interaction in the breast's genomic signature of pregnancy. Sci Rep 2021;11:2643. 33514799 10.1038/s41598-021-81704-8 PMC7846553

[30] Russo J . The future of prevention and treatment of breast cancer. Springer; 2021.

[31] Russo J , Russo IH. DNA labeling index and structure of the rat mammary gland as determinants of its susceptibility to carcinogenesis. J Natl Cancer Inst 1978;61:1451–9. 102857

[32] Russo J , Russo IH. Influence of differentiation and cell kinetics on the susceptibility of the rat mammary gland to carcinogenesis. Cancer Res 1980;40:2677–87. 7388818

[33] Yaghjyan L , Austin-Datta RJ, Oh H, Heng YJ, Vellal AD, Sirinukunwattana K, . Associations of reproductive breast cancer risk factors with breast tissue composition. Breast Cancer Res 2021;23:70. 34225771 10.1186/s13058-021-01447-2 PMC8258947

[34] Heusinger K , Loehberg CR, Haeberle L, Jud SM, Klingsiek P, Hein A, . Mammographic density as a risk factor for breast cancer in a German case-control study. Eur J Cancer Prev 2011;20:1–8. 21102341 10.1097/CEJ.0b013e328341e2ce

[35] Yaghjyan L , Colditz GA, Rosner B, Bertrand KA, Tamimi RM. Reproductive factors related to childbearing and mammographic breast density. Breast Cancer Res Treat 2016;158:351–9. 27351801 10.1007/s10549-016-3884-y PMC5527672

[36] Alexeeff SE , Odo NU, McBride R, McGuire V, Achacoso N, Rothstein JH, . Reproductive factors and mammographic density: associations among 24,840 women and comparison of studies using digitized film-screen mammography and full-field digital mammography. Am J Epidemiol 2019;188:1144–54. 30865217 10.1093/aje/kwz033 PMC6545283

[37] Mariapun S , Li J, Yip CH, Taib NAM, Teo S-H. Ethnic differences in mammographic densities: an Asian cross-sectional study. PLoS One 2015;10:e0117568. 25659139 10.1371/journal.pone.0117568 PMC4320072

[38] Hjerkind KV , Ellingjord-Dale M, Johansson ALV, Aase HS, Hoff SR, Hofvind S, . Volumetric mammographic density, age-related decline, and breast cancer risk factors in a national breast cancer screening program. Cancer Epidemiol Biomarkers Prev 2018;27:1065–74. 29925631 10.1158/1055-9965.EPI-18-0151

[39] Vandeloo MJ , Neven KY, Bruckers LM, Russo J, Vancoillie L, Bijnens EM, . Abstract PS7-32: age of first full term pregnancy and other reproductive factors affect mammographic breast density in postmenopausal women. Cancer Res 2021;81:PS7-32. 10.1158/2767-9764.CRC-24-0561 PMC11803437 39835404

[40] Rice MS , Tamimi RM, Bertrand KA, Scott CG, Jensen MR, Norman AD, . Does mammographic density mediate risk factor associations with breast cancer? An analysis by tumor characteristics. Breast Cancer Res Treat 2018;170:129–41. 29502324 10.1007/s10549-018-4735-9 PMC5994184

[41] Ward SV , Burton A, Tamimi RM, Pereira A, Garmendia ML, Pollan M, . The association of age at menarche and adult height with mammographic density in the International Consortium of Mammographic Density. Breast Cancer Res 2022;24:49. 35836268 10.1186/s13058-022-01545-9 PMC9284807

[42] Ghadge AG , Dasari P, Stone J, Thompson EW, Robker RL, Ingman WV. Pubertal mammary gland development is a key determinant of adult mammographic density. Semin Cell Dev Biol 2021;114:143–58. 33309487 10.1016/j.semcdb.2020.11.011

[43] Kim S , Mai Tran TX, Kim MK, Chung MS, Lee EH, Lee W, . Associations between breast cancer risk factors and mammographic breast density in a large cross-section of Korean women. Eur J Cancer Prev 2024;33:407–13. 38375880 10.1097/CEJ.0000000000000878

[44] Azam S , Sjölander A, Eriksson M, Gabrielson M, Czene K, Hall P. Determinants of mammographic density change. JNCI Cancer Spectr 2019;3:pkz004. 31360892 10.1093/jncics/pkz004 PMC6649843

[45] Park B , Lim SE, Ahn H, Yoon J, Choi YS. Heterogenous effect of risk factors on breast cancer across the breast density categories in a Korean screening population. Cancers (Basel) 2020;12:1391. 32481621 10.3390/cancers12061391 PMC7352951

[46] Hunt JT , Kamat R, Yao M, Sharma N, Batur P. Effect of contraceptive hormonal therapy on mammographic breast density: a longitudinal cohort study. Clin Imaging 2023;97:62–7. 36893493 10.1016/j.clinimag.2023.03.001

[47] Azam S , Jacobsen KK, Aro AR, Lynge E, Andersen ZJ. Hormone replacement therapy and mammographic density: a systematic literature review. Breast Cancer Res Treat 2020;182:555–79. 32572713 10.1007/s10549-020-05744-w PMC7320951

[48] Lee HN , Sohn YM, Han KH. Comparison of mammographic density estimation by Volpara software with radiologists’ visual assessment: analysis of clinical-radiologic factors affecting discrepancy between them. Acta Radiol 2015;56:1061–8. 25338836 10.1177/0284185114554674

