# Supplementary material for: Age at First Full-term Pregnancy and Other Reproductive Factors Are Associated with Mammographic Breast Density in Postmenopausal Women: A Study in Flanders, Belgium
Source: Cancer Res Commun. 2025 Feb 7;5(2):267–76. doi: 10.1158/2767-9764.CRC-24-0561 (PMC11803437; doi:10.1158/2767-9764.CRC-24-0561)
Supplement: Table S1 — Results from the univariate analysis for the glandular tissue (GLAND). For FFTP, we used the proc NLIN procedure, for the others a GLM. Multiplicative estimates provided are calculated according to eβ for the given unit change [file crc-24-0561_table_s1_suppst1.docx]

**Supplementary Table S1**

**Table S1**: Results from the univariate analysis for the glandular tissue (GLAND). For FFTP, we used the proc NLIN procedure, for the others a GLM. Multiplicative estimates provided are calculated according to e^β^ for the given unit change

| **Variable** | Estimate | 95%CI LL | 95%CI UL | p-value |
| --- | --- | --- | --- | --- |
| **FFTP***, +1 year* |  |  |  |  |
| *≤ 25.7* | -0.29% | -2.70% | 2.18% | 0.83 |
| *> 25.7* | 1.84% | 0.28% | 3.43% | 0.021 |
| **Age at MBD measurement***, +1 year* | -1.10% | -1.72% | -0.47% | 0.0006 |
| **Age at the menarche***, + 1 year* | 6.22% | 3.67% | 8.83% | <0.0001 |
| **Use of a contraception pill***, yes compared to no* | -4.83% | -16.13% | 8.00% | 0.44 |
| **Use of hormones during menopause***, yes compared to no* | 10.85% | 1.48% | 21.09% | 0.0223 |
| **Current BMI (kg/m²)** |  |  |  |  |
| **Underweight and normal** | Referent |  |  |  |
| **Overweight** | -38.82 | -42.60 | -34.80 | <0.0001 |
| **Obese** | -58.01 | -61.19 | -54.57 | <0.0001 |
| **Number of liveborn children** |  |  |  |  |
| **One child** | Referent |  |  |  |
| **Two children** | 2.37% | -6.93% | 12.59% | 0.63 |
| **Three or more children** | -5.15% | -13.81% | 4.38% | 0.28 |

Abbreviations: BMI = body mass index; CI = confidence interval; FFTP = first full-term pregnancy, MBD = mammographic breast density: NLIN = non linear regression
