# Supplementary material for: Age at First Full-term Pregnancy and Other Reproductive Factors Are Associated with Mammographic Breast Density in Postmenopausal Women: A Study in Flanders, Belgium
Source: Cancer Res Commun. 2025 Feb 7;5(2):267–76. doi: 10.1158/2767-9764.CRC-24-0561 (PMC11803437; doi:10.1158/2767-9764.CRC-24-0561)
Supplement: Table S2 — Results from the univariate analysis for the volumetric breast density (VBD). For FFTP, we used the proc NLIN procedure, for the others a GLM. Multiplicative estimates provided are calculated according to eβ for the given unit change [file crc-24-0561_table_s2_suppst2.docx]

**Supplementary Table S2**

**Table S2**: Results from the univariate analysis for the volumetric breast density (VBD). For FFTP, we used the proc NLIN procedure, for the others a GLM. Multiplicative estimates provided are calculated according to e^β^ for the given unit change

| **Variable** | | Estimate | | 95%CI LL | | 95%CI UL | | p-value | |
| --- | --- | --- | --- | --- | --- | --- | --- | --- | --- |
| **FFTP***, +1 year* | |  | |  | |  | |  | |
| *≤ 25.7* | | -0.66% | | -3.07% | | 1.82% | | 0.36 | |
| *> 25.7* | | 2.15% | | 0.58% | | 3.75% | | 0.007 | |
| **Age at MBD measurement***, +1 year* | | -1.11% | | -1.74% | | -0.49% | | 0.0005 | |
| **Age at the menarche***, + 1 year* | | 5.98% | | 3.42% | | 8.60% | | <0.0001 | |
| **Use of a contraception pill***, yes compared to no* | | -5.02% | | -16.36% | | 7.85% | | 0.43 | |
| **Use of hormones during menopause***, yes compared to no* | | 9.74% | | 0.41% | | 19.94% | | 0.040 | |
| **Current BMI (kg/m²)** | |  | |  | |  | |  | |
| **Underweight and normal** | | Referent | |  | |  | |  | |
| **Overweight** | | -38.19% | | -42.05% | | -34.07% | | <0.0001 | |
| **Obese** | | -57.80% | | -61.03% | | -54.29% | | <0.0001 | |
| **Number of liveborn children** | |  | |  | |  | |  | |
| **One child** | | Referent | |  | |  | |  | |
| **Two children** | | 0.16% | | -8.98% | | 10.21% | | 0.97 | |
| **Three or more children** | | -8.38% | | -16.78% | | 0.87% | | 0.075 | |

Abbreviations: BMI = body mass index; CI = confidence interval; FFTP = first full-term pregnancy, MBD = mammographic breast density: NLIN = non linear regression
