# Supplementary material for: Age at First Full-term Pregnancy and Other Reproductive Factors Are Associated with Mammographic Breast Density in Postmenopausal Women: A Study in Flanders, Belgium
Source: Cancer Res Commun. 2025 Feb 7;5(2):267–76. doi: 10.1158/2767-9764.CRC-24-0561 (PMC11803437; doi:10.1158/2767-9764.CRC-24-0561)
Supplement: Table S3 — Results from the univariate ordinal logistic regression model for BI-RADS. The model for FFTP was stratified based on the results from the GLAND and VBD models: ‘≤ 25.7 years’ and ‘> 25.7 years’. Data is presented as Odds Ratio (OR) estimates and 95% Wald confidence intervals [file crc-24-0561_table_s3_suppst3.docx]

**Supplementary Table S3**

**Table S3:** Results from the univariate ordinal logistic regression model for **BI-RADS**®. The model for FFTP was stratified based on the results from the GLAND and VBD models: ‘≤ 25.7 years’ and ‘> 25.7 years’. Data is presented as Odds Ratio (OR) estimates and 95% Wald confidence intervals

|  |  | **For an FFTP *≤* 25.7 years**  **(n = 504)** | | | |  | **For an FFTP > 25.7 years**  **(n=530)** | | | |
| --- | --- | --- | --- | --- | --- | --- | --- | --- | --- | --- |
| **Variable** |  | OR | 95%CI LL | 95%CI UL | p-value |  | OR | 95%CI LL | 95%CI UL | p-value |
| **FFTP***, +1 year* |  | 0.99 | 0.91 | 1.07 | 0.72 |  | 1.03 | 0.98 | 1.09 | 0.18 |
| **Age at MBD measurement***, +1 year* |  | 0.99 | 0.96 | 1.02 | 0.53 |  | 0.96 | 0.94 | 0.99 | 0.0123 |
| **Age at the menarche***, + 1 year* |  | 1.24 | 1.11 | 1.39 | 0.0002 |  | 1.17 | 1.04 | 1.31 | 0.0068 |
| **Use of a contraception pill***, yes compared to no* |  | 0.91 | 0.54 | 1.53 | 0.73 |  | 0.71 | 0.37 | 1.38 | 0.31 |
| **Use of hormones during menopause***, yes compared to no* |  | 1.22 | 0.83 | 1.80 | 0.32 |  | 1.79 | 1.18 | 2.73 | 0.0067 |
| **Current BMI (kg/m²)** |  |  |  |  |  |  |  |  |  |  |
| **Underweight and normal** |  | Ref |  |  |  |  |  |  |  |  |
| **Overweight** |  | 0.17 | 0.11 | 0.25 | 0.29 |  | 0.20 | 0.14 | 0.30 | 0.86 |
| **Obese** |  | 0.04 | 0.02 | 0.07 | <0.0001 |  | 0.04 | 0.02 | 0.07 | <0.0001 |
| **Number of liveborn children** |  |  |  |  |  |  |  |  |  |  |
| **One child** |  | Ref |  |  |  |  |  |  |  |  |
| **Two children** |  | 0.81 | 0.52 | 1.25 | 0.94 |  | 1.16 | 0.76 | 1.78 | 0.0437 |
| **Three or more children** |  | 0.67 | 0.43 | 1.05 | 0.09 |  | 0.68 | 0.44 | 1.05 | 0.0068 |

Abbreviations: BMI = body mass index; CI = confidence interval; FFTP = first full-term pregnancy, MBD = mammographic breast density; OR = odds ratio
